# Purity and Enrichment of Laser-Microdissected Midbrain Dopamine Neurons

**DOI:** 10.1155/2013/747938

**Published:** 2013-07-25

**Authors:** Amanda L. Brown, Trevor A. Day, Christopher V. Dayas, Doug W. Smith

**Affiliations:** ^1^Neurobiology of Ageing Laboratory, School of Biomedical Sciences and Pharmacy, Centre for Translational Neuroscience and Mental Health Research, University of Newcastle, Newcastle, NSW 2308, Australia; ^2^Hunter Medical Research Institute, Kookaburra Circuit, New Lambton Heights, Newcastle, NSW 2305, Australia; ^3^Faculty of Science, Engineering & Built Environment, Waurn Ponds Campus, Locked Bag 20000, Geelong, VIC 3220, Australia

## Abstract

The ability to microdissect individual cells from the nervous system has enormous potential, as it can allow for the study of gene expression in phenotypically identified cells. However, if the resultant gene expression profiles are to be accurately ascribed, it is necessary to determine the extent of contamination by nontarget cells in the microdissected sample. Here, we show that midbrain dopamine neurons can be laser-microdissected to a high degree of enrichment and purity. The average enrichment for tyrosine hydroxylase (TH) gene expression in the microdissected sample relative to midbrain sections was approximately 200-fold. For the dopamine transporter (DAT) and the vesicular monoamine transporter type 2 (Vmat2), average enrichments were approximately 100- and 60-fold, respectively. Glutamic acid decarboxylase (Gad65) expression, a marker for GABAergic neurons, was several hundredfold lower than dopamine neuron-specific genes. Glial cell and glutamatergic neuron gene expression were not detected in microdissected samples. Additionally, SN and VTA dopamine neurons had significantly different expression levels of dopamine neuron-specific genes, which likely reflects functional differences between the two cell groups. This study demonstrates that it is possible to laser-microdissect dopamine neurons to a high degree of cell purity. Therefore gene expression profiles can be precisely attributed to the targeted microdissected cells.

## 1. Introduction

The midbrain dopamine system, comprising the nigrostriatal, mesocortical, and mesolimbic pathways, is involved in many brain functions, such as motor control, cognition, and reward behaviors [1]. Dysfunction or degeneration of midbrain dopamine neurons is associated with a number of neurological conditions including Parkinson's disease (PD), cognitive impairment, and addiction. One way to probe the mechanisms associated with both normal and abnormal functions of the dopamine system is to characterize the molecular profile of dopamine neurons themselves. The advent of laser microdissection has made this possible. For example, dopamine neurons have been microdissected, and gene expression profiles have been compared between ventral tegmental area (VTA) subpopulations [2] and between dopamine and other catecholaminergic neurons [3]. However, while various neuronal identification and laser microdissection methods have been employed to segregate dopamine neurons from surrounding brain tissue, the critical issue of sample purity has not been adequately addressed.

There is considerable cellular heterogeneity within the substantia nigra (SN) and VTA regions of the midbrain. Stereological estimates of GABA and dopamine neuron populations in SN and VTA indicate that there are more GABA than dopamine neurons, and in some subregions there are nearly three times as many [4]. Additionally, in the VTA, glutamate neurons intermingle with the dopamine neuron population [4, 5]. Therefore, there is a very real chance for cross-contamination during microdissection of targeted dopamine neurons. At a minimum, the degree to which the targeted population is contaminated with unwanted cells or cell fragments must be known if meaningful interpretations of molecular profiles are to be made. This is particularly important when the gene(s) of interest are expressed at relatively low levels in the target population (e.g., dopamine neurons) but are relatively highly expressed in surrounding cells that might be inadvertently included in the microdissected sample (e.g., GABA or glutamate neurons).

Recent studies have demonstrated the potential of dopamine neuron microdissection. For example, Liss and colleagues were able to distinguish midbrain dopamine neuron subpopulations on the basis of tyrosine hydroxylase (TH), vesicular monoamine transporter type 2 (Vmat2), and dopamine transporter (DAT) transcript expression ratios [2] and, in a separate study, demonstrated increased *α*-synuclein transcript levels in SN dopamine neurons from PD brains [6]. Such comparisons could not be readily made without microdissection. However, in neither of these studies was the extent of contamination by nondopamine cells adequately determined [2, 6]. The molecular analysis for cell purity was qualitative and insensitive compared to quantitative polymerase chain reaction (qPCR) methods. Also, the apparent purity may have resulted from the relative homogeneity of the target SNpc dopamine group, where dopamine neurons outnumber GABA neurons by nearly threefold [4]. Furthermore, SNpc dopamine neurons are relatively large and easy to visually differentiate, even with Nissl, a noncell-specific label. In contrast, VTA dopamine neuron subpopulations consist of cells not readily identifiable on the basis of size and location. Therefore, if stringent analyses are required for these subpopulations [7], reliable and accurate identification of dopamine neurons through immunolabeling for a dopamine-specific marker, such as TH, is necessary.

We have previously reported a method that permits the extraction of high quality RNA from immunolabeled, microdissected cells [8]. The primary aim of the present study was to assess the cellular purity of laser-microdissected dopamine neurons that were obtained following immunolabeling for TH using this method of RNA preservation. Our results show that relatively large numbers of dopamine neurons can be isolated from the surrounding tissue to produce a sample that contains a highly enriched and relatively pure population of dopamine neurons. In addition, we compared the molecular profiles of SN and VTA dopamine neurons.

## 2. Materials and Methods

### 2.1. Tissue Immunolabeling and Preparation for Laser Microdissection

All animal work was carried out in strict accordance with institutional, state, and national animal ethics regulations. Tissue preparation and identification of midbrain dopamine neurons by TH immunofluorescence was carried out essentially as described [8]. Briefly, ten micron thick midbrain cryosections, archived at –80°C, were rinsed in PBS (15 secs) and then fixed in acetone (5 min). Acetone was removed by a rapid PBS rinse and the slide placed in 2 M NaCl PBS. Sections were incubated overnight at 4°C with mouse anti-TH monoclonal antibody (MAB318, Chemicon, Millipore), diluted 1 : 50 in 2 M NaCl PBS. Unbound primary antibody was removed by rinsing with 2 M NaCl in PBS (5 min). Sections were then incubated overnight at 4°C with Alexa Fluor 488 conjugated donkey anti-mouse (Invitrogen), at 1 : 100 in 2 M NaCl PBS. Unbound secondary antibody was removed by a 2 M NaCl PBS rinse. Immediately prior to microdissection, excess NaCl was removed by a rapid PBS rinse, and sections were then dehydrated twice in 100% ethanol (1 min each) and then delipidated twice in xylene (2 min each). With the exception of the room temperature ethanol and xylene, all solutions were ice-cold. All PBS solutions were diethyl pyrocarbonate (DEPC) treated.

### 2.2. Laser Microdissection and RNA Extraction

Laser microdissection and pressure catapulting of dopamine neurons were carried out using a PALM MicroBeam system (Zeiss). Briefly, TH immunolabeled midbrain dopamine neurons were visualised with the system's Axiovert 200 M inverted fluorescent microscope. The UVA laser was then focused in the plane of the tissue section, and the cell body was separated from surrounding tissue using laser ablation to remove a thin line of tissue at the cell body border. Removal of the cell body from the glass slide was accomplished through defocussing the laser beam and pressure catapulting cells into lysis buffer contained in a PCR tube lid, mounted directly above the cells of interest. For both laser cutting and catapulting, the minimum amount of laser energy was used to achieve separation of the cell from its surrounding tissue (laser cutting) and for removal of the cell from the microscope slide (laser catapulting). For each animal a total of three hundred dopamine cell bodies from each of the SN and VTA were UVA laser microdissected. To minimize the length of time tissue sections were held on the microdissection microscope stage, and cells were pressure catapulted into 30 *μ*L RLT lysis buffer (RNeasy, Qiagen) in lots of 100. Following cell collection, lysis buffer volume was increased to 100 *μ*L, and the sample homogenized by vortexing and then placed at −80°C for later use. Prior to RNA extraction, the three 100 dopamine neuron samples were combined, and total RNA was extracted using the RNeasy microkit (Qiagen) according to manufacturer's instructions, including addition of poly-A carrier RNA to the lysate and an on-column DNAse-I treatment.

### 2.3. Reverse Transcription and qPCR of Laser-Microdissected Sample

Reverse transcription was carried out using SuperScript III (Invitrogen), according to manufacturer's instructions and as previously described [8]. Two-thirds of the total RNA extracted was used in a 20 *μ*L RT+ cDNA synthesis reaction, with the remaining third used in the RT− reaction. One microliter of cDNA product was added to each qPCR reaction, with the qPCR protocol, and all other reagents and volumes as described [8]. Primer sequences are listed in Table 1. The entire process of dopamine neuron laser microdissection and qPCR was carried out three independent times. qPCR reactions were run in triplicate for each gene. The 18S ribosomal RNA gene was chosen for normalization and the delta Ct ((ΔCt) threshold cycle) was determined for each gene relative to 18S. The ΔΔCt method [9] was used for gene expression comparisons between whole midbrain sections and laser-microdissected dopamine neurons and between SN and VTA dopamine populations.

### 2.4. RNA Extraction, Reverse Transcription, and qPCR of Whole Midbrain Sections

To enable assessment of dopamine neuron enrichment conferred by microdissection, total RNA from whole midbrain sections (containing dopamine neurons) was extracted, contaminating genomic DNA removed by on-column DNase-I digestion using Qiagen's RNeasy microkit and DNase reagents according to manufacturer's instructions. The concentration of total RNA was determined using Thermo Scientific NanoDrop 1000 spectrophotometer. Reverse transcription and qPCR were carried out as described above, with 60 ng of RNA added to 20 *μ*L RT+ and RT− cDNA synthesis reactions and 1 ng of cDNA added to each qPCR reaction.

### 2.5. Statistical Analysis

To assess purity and enrichment of the microdissected population, a comparison between whole midbrain sections and either SN or VTA laser microdissected dopamine neurons was made using an unpaired Student's *t*-test for each gene. A comparison of the SN and VTA dopamine neuron molecular profiles was also statistically analyzed using an unpaired Student's *t*-test. An alpha level of .05 was applied for all statistical tests.

## 3. Results

### 3.1. Purity and Enrichment of Laser-Microdissected Midbrain Dopamine Neurons

**Figure 1:**
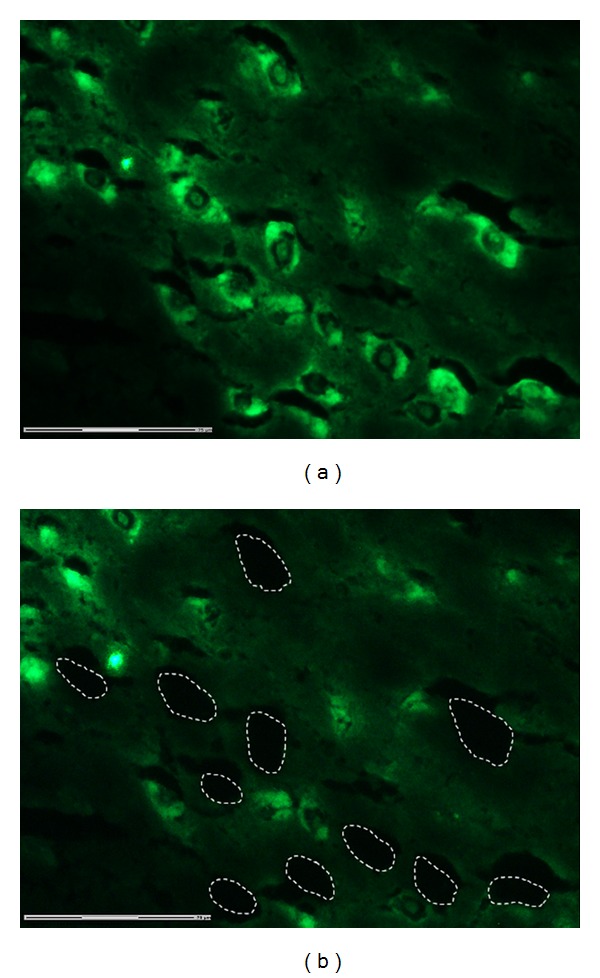
Ventral midbrain showing dopamine neurons identified by tyrosine hydroxylase immunofluorescent labeling. Fresh-frozen brains were cryosectioned at 10 microns, acetone fixed, and immunolabeled in high salt buffer, prior to dehydration and delipidation in preparation for laser microdissection using a Zeiss LMD system. (a) Prior to microdissection. (b) Following selection and laser microdissection of several immunofluorescent dopamine neurons. Dashed outlines indicate where the dopamine cell bodies were laser-captured from. A number of labeled dopamine neurons remain for reference.

As is evident from Figure 1 and consistent with our previous work [8], midbrain dopamine neurons, immunofluorescently labeled using high salt buffer for RNA preservation, were readily identified and laser microdissected from 10 micron thick sections. Approximately 300 SN and 300 VTA dopamine cell bodies were collected for downstream molecular analysis for each experiment. RNA was also extracted from dopamine neuron-containing, whole midbrain sections located on the same microscope slide as sections used for dopamine neuron microdissection. This allowed enrichment and purity assessments and RNA quality controls to be carried out.

**Figure 2:**
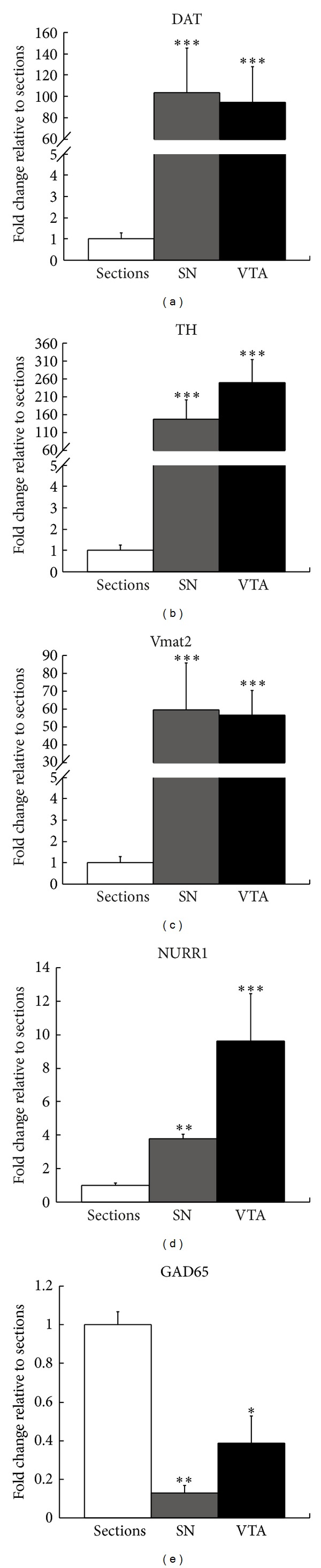
Assessment of dopamine neuron sample enrichment and purity by qPCR. The graph column title, “Sections,” refers to midbrain sections that have not been altered in terms of cellular content and therefore contain dopamine neurons and many other cell types. They constitute an appropriate reference for the estimation of enrichment and purity of a population of cells isolated from midbrain sections. Increased expression of the dopaminergic genes: DAT (a), TH (b), Vmat2 (c), and NURR1 (d), in SN and VTA laser-microdissected samples relative to whole midbrain sections is indicative of dopamine neuron enrichment. A significant reduction in expression of the GABA gene, GAD65 (e), and the lack of detection of the genes encoding for vesicular glutamate transporter 2 (Vglut2), and the glial gene, Gfap (data not shown), within the SN and VTA suggests isolation of a relatively pure population of dopamine neurons. The Ct for each gene was normalized to the Ct for the 18S rRNA reference gene, and data are presented as mean fold change in expression relative to sections ± SEM. Asterisks indicate significant difference to whole midbrain sections. *P ≤ 0.05, **P ≤ 0.01, and ***P ≤ 0.001; *n* = 3.

To determine the level of purity of the microdissected cells, relative expression levels for dopamine-related and nondopamine-related genes were quantified. The dopamine neuron-specific genes were those encoding the dopamine transporter (Slc6a3/DAT), tyrosine hydroxylase (TH), vesicular monoamine transporter type 2 (Slc18a2/Vmat2), and the nuclear orphan receptor (Nr4a2/NURR1). The nondopamine neuron-specific genes were the glutamate neuron-specific vesicular glutamate transporter type 2 (Slc17a6/Vglut2), the GABA neuron-specific glutamic acid decarboxylase 65 (Gad2/Gad65), and the glial cell-specific glial fibrillary acidic protein (Gfap). The relative expression levels of all dopamine neuron-related transcripts were significantly increased in the SN and VTA microdissected dopamine neurons compared to whole sections, with DAT being 104- and 95-, TH 147- and 249-, Vmat2 60- and 57-, and NURR1 4- and 10-fold enriched in SN and VTA, respectively (Figure 2). Neither expression of the glutamate neuron gene, Slc17a6/Vglut2, nor that of the glial gene, Gfap, was detected in either the SN or VTA microdissected dopamine neuron samples. In contrast, expression of the GABA neuron gene, Gad65, was detected in both the SN and VTA microdissected dopamine neuron samples (Figure 2). However, its abundance was relatively low compared to dopamine neuron-specific genes. For example, Gad65 in both the SN and VTA microdissected dopamine neuron samples was several hundredfold less abundant than TH.

### 3.2. Comparison of SN and VTA Dopamine Neuron Expression Profiles

**Figure 3:**
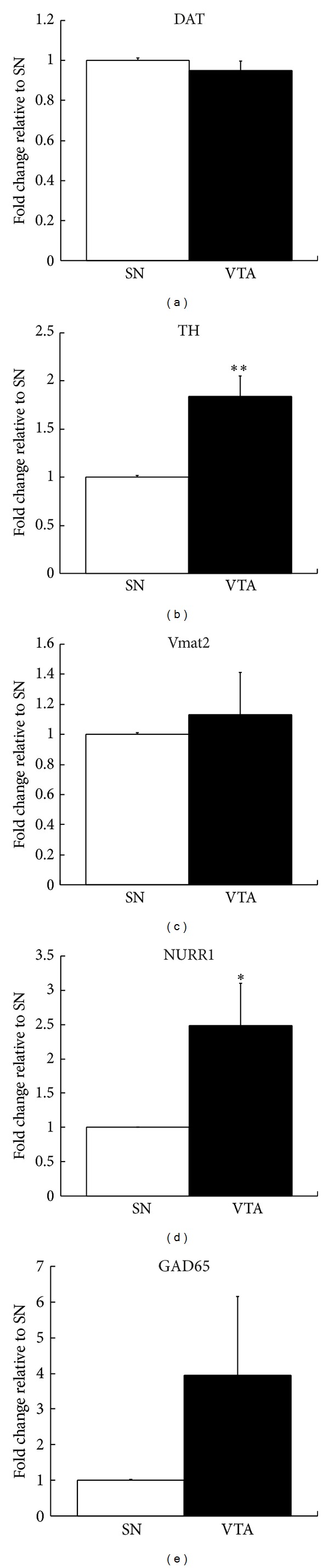
The relative gene expression profiles of laser-microdissected SN and VTA dopamine neurons. Expression levels were compared using qPCR for dopamine neuron genes: DAT (a); TH (b); Vmat2 (c); NURR1 (d); and the GABA neuron gene, GAD65 (e). Note, significantly different expression levels were only found for TH and NURR1 transcripts, where there was an increase in VTA microdissected dopamine neurons relative to SN. The Ct for each gene was normalized to the Ct for the 18S rRNA reference gene, and data are presented as mean fold change in expression relative to SN ± SEM. Asterisks indicate significant difference relative to SN. *P ≤ 0.05, **P ≤ 0.01, and ***P ≤ 0.001; *n* = 3.

**Figure 4:**
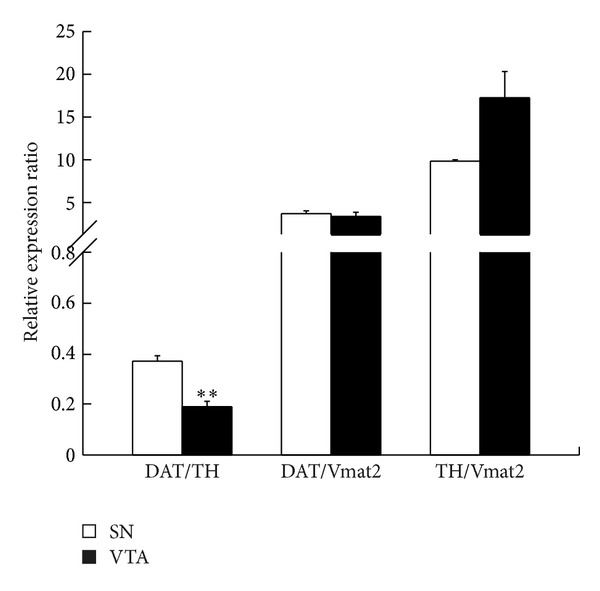
Coexpression patterns of DAT, TH, and Vmat2 in SN and VTA dopamine neurons. VTA dopamine neurons display a significantly lower DAT/TH expression ratio compared to their SN counterparts. However, there were no significant differences in DAT/Vmat2 or TH/Vmat2 between the two dopamine neuron populations. There was a trend towards a significantly higher TH/Vmat2 ratio in VTA dopamine neurons (*P* = 0.06). Data are expressed as mean ΔΔCt ± SEM, where the ΔCt of each gene in each region is normalized to 18S. ΔΔCt is then determined between the normalized genes for each region. Asterisks indicate significant difference relative to SN. *P ≤ 0.05, **P ≤ 0.01; *n* = 3.

The midbrain SN and VTA dopamine neuron populations are, for the most part, anatomically separate. It is possible, therefore, to microdissect the dopamine neurons of the two populations and carry out a comparative analysis. Firstly, we compared expression levels of the dopamine neurotransmission-related genes (TH, DAT, and Vmat2) within each of the two populations and for both the SN and VTA. TH expression was significantly higher than DAT (*P* < 0.01) and DAT expression significantly higher than Vmat2 (*P* < 0.01), and this was the case for both SN and VTA. The relative expression for each gene between the SN and VTA was then compared.  Figure 3 shows there was no significant difference in expression of DAT and Vmat2 between the SN and VTA dopamine neurons. However, for TH and NURR1, the VTA dopamine neurons showed a 1.8- and 2.5-fold increase over SN dopamine neurons, respectively. Lastly, the relative expression ratios for dopamine neuron-specific transcripts were compared between the SN and VTA. The DAT/TH ratio was significantly lower in VTA compared to SN dopamine neurons (Figure 4; *P* < 0.01), there was no significant difference in the DAT/Vmat2 ratio between the two regions (Figure 4; *P* = 0.57), and there was a trend toward a higher TH/Vmat2 ratio in the VTA (*P* = 0.06).

## 4. Discussion

The ability to microdissect specific cells from complex, heterogeneous tissues, such as the brain, is advantageous for transcriptomics as it limits a potential artifact, caused by dilution effects, that is characteristic of studies using tissue homogenates as starting material. However, if meaningful interpretations of transcriptomic investigations of specific cell types or single cells are to be obtained, two important issues need to be considered. These include sample RNA integrity and the degree of contamination with untargeted cells or cell fragments. We have previously developed and demonstrated a method that preserves RNA integrity during the immunolabeling process that is generally obligatory for the identification of cells for microdissection [8]. In the present study, we addressed the issue of microdissected sample purity. Using our protocol for preserving RNA quality, we were able to isolate an enriched and relatively pure population of midbrain dopamine neurons. Furthermore, as this method also allows for the identification and collection of anatomically distinct subpopulations, a comparison of gene expression between the SN and VTA dopamine neurons was also made.

To assess the level of enrichment for dopamine neurons in microdissected samples, dopamine neuron-associated gene expression was compared to that in whole midbrain sections. The degree of enrichment reflects the relative number of target and nontarget cells within the section being microdissected and the efficiency of the microdissection process in terms of its ability to quantitatively capture all the targeted cells. As predicted, expression levels of the dopamine neuron-specific genes, DAT, TH, and Vmat2, and the dopamine neuron-associated gene NURR1, were all found to be markedly increased, relative to the 18S reference gene, in microdissected dopamine neurons compared to whole midbrain sections, and this was the case in both the SN and VTA. In comparison to DAT, TH, and Vmat2, the enrichment of NURR1 was modest, presumably reflecting its lack of absolute specificity for dopamine neurons in the midbrain [10, 11]. Importantly, this demonstration of marked enrichment underscores how readily cell-specific transcript information could be lost if molecular profiling experiments are carried out on tissue homogenates.

To be able to accurately attribute changes in gene expression to a particular cell or cell type, it is important to determine the purity of the sample. Contamination of microdissected dopamine neuron samples by glial cell, glutamate, and GABA neurons was assessed by qPCR for the presence of Gfap, Vglut2, and Gad65 transcripts, respectively. Transcripts for either Gfap or Vglut2 were not detected in microdissected dopamine neuron samples, indicating that contamination by either glial or glutamate cells did not occur to a significant level. However, a small but significant contamination by GABA neurons was found, with Gad65 transcripts being detected in microdissected dopamine neuron samples from both the SN and VTA. The level of GABA transcript contamination was extremely low, being approximately 500-fold less than the average for dopamine neuron-specific TH transcripts in the SN, and it was not reliably detected in all samples as the qPCR Ct value was typically close to 40. The level of GABA neuron contamination was higher in the VTA samples, which could have resulted from an increased difficulty to microscopically visualize, select, and extract often smaller more clustered VTA dopamine neurons during the laser microdissection process [2]. The source of contamination is most likely from GABA neurons partially overlapping the dopamine cells. During microdissection, the target cell is separated from adjoining tissue by laser ablation, and we directed the laser precisely to the dopamine cell body boundary to minimize collection of adjacent nondopamine cells during subsequent laser catapulting. However, while we minimized the potential for contamination by overlapping cells by using 10 micron thick cryosections, the presence of Gad65 transcripts in the microdissected sample indicates that this approach was not completely effective. Thinner cryosections may resolve this contamination issue. 

Our lack of evidence for Vglut2 transcripts in microdissected dopamine neuron samples is somewhat surprising given the neuroanatomical [5, 12, 13], electrophysiological [14, 15], genetic/optogenetic [16–18], and behavioral reports [19], indicating that at least some midbrain dopamine neurons are capable of glutamate cotransmission (for review see [20]). However, the extent of dopamine and glutamate coexistence as neurotransmitters in midbrain dopamine neurons is limited, with SN being devoid of Vglut2 and dorsal striatum corelease [12, 17]. The lack of detection of Vglut2 transcripts in our microdissected SN dopamine neuron samples is consistent with these findings. With regard the VTA, Vglut2 coexpressing dopamine neurons are mostly found rostromedially, particularly in the rostral linear subgroup [12]. As we microdissected a relatively small number (300) of the total VTA dopamine neuron population (~40,000 cells, [4]) from representative sections throughout the rostrocaudal extent of the VTA, it is possible that Vglut2 transcripts were too diluted for detection. Additionally, the expression of the glutamatergic phenotype in midbrain dopamine neurons appears to be developmentally regulated, with essentially nonexistent coexpression of TH and Vglut2 in either SN or VTA of 90-day-old rats [21] and a marked reduction in mice between P0 and P45 [22]. As our rats were 4-5 months old, it is likely that there would be minimal residual Vglut2 expression in VTA dopamine neurons. 

One advantage of the immunolaser-microdissection technique used in the present study is that it can precisely isolate individual cells. Therefore, it was possible to accurately collect the anatomically segregated dopamine neurons of the SN and VTA. It is well established that dopamine neurons of the VTA and SN and subpopulations within these nuclei are similar with respect to the dopamine phenotype, but distinct with regard to other properties, such as their ability to regulate calcium [23], respond to opioids [24], and coexpress glutamate as a neurotransmitter [12]. The ability to investigate the various subpopulations of dopamine neurons is particularly important for studies of disease states. Consistent with inter- and intranuclei differences, we found TH and NURR1 expression to be significantly higher in VTA relative to SN dopamine neurons but no differences in DAT or Vmat2 expression levels (Figure 3). Furthermore, the relative expression levels of components of the neurotransmission pathway in dopamine neurons, namely, TH for synthesis, Vmat2 for vesicular packaging, and DAT for cellular reuptake, can be used to infer cell function. For example, increased dopamine synthesis relative to cellular dopamine reuptake that is a low DAT/TH ratio could imply relatively long transmission duration, consistent with the known neuromodulatory, or volume transmission, role of this neurotransmitter [2]. It has previously been shown that subpopulations of dopamine neurons can display distinct patterns of dopamine gene coexpression [2]. Using a comparative molecular analysis of projection target-defined, midbrain dopamine neurons, Lammel and colleagues demonstrated a significant variation in DAT/TH and DAT/Vmat2 expression ratios across the midbrain dopamine subgroups and showed that the differences were due to varying levels of DAT expression. All VTA subgroups had greater levels of TH compared to DAT, and Vmat2 expression was consistently the lowest of the three dopamine genes [2]. In the present study, we found TH to be the most highly expressed dopamine gene transcript, in both the SN and VTA, with DAT having the second highest level of expression and Vmat2 the lowest. Furthermore, we found the ratios of DAT to TH to differ between the SN and VTA, with there being much less DAT relative to TH in VTA compared to SN dopamine neurons (Figure 4). This pattern of a greater relative level of DAT compared to TH in SN is consistent with Lammel's findings in the mouse. Unlike Lammel, we did not find a significant difference in DAT/Vmat2 between the SN and VTA dopamine neurons. Another significant difference between the Lammel and present studies is that their ratio variances were due to DAT expression differences, whereas ours are due to TH. When DAT expression is compared between the two regions, there is no significant difference, unlike TH expression, which is significantly higher in VTA dopamine neurons (Figure 3). This probably is also why we found no difference in the DAT/Vmat2 ratios between the SN and VTA but a trend towards a significant difference in TH/Vmat2 ratios. These differences between the two studies likely reflect interspecies variations.

## 5. Conclusions

By combining an immunofluorescent labeling method with laser microdissection to specifically target midbrain dopamine neurons, this study demonstrates that large numbers of these neurons can be extracted from separate cell groups to produce samples enriched and relatively pure for SN and VTA dopamine neurons. Indeed, this method could readily be combined with existing tract tracing techniques to allow for greater dopamine neuron subpopulation analysis. As the method employed an immunolabeling protocol designed to preserve high quality RNA, it also allows for the meaningful interpretation of downstream molecular analysis.

## Figures and Tables

**Table 1:** Sequences for qPCR primers.

Gene name	Gene ID	Forward primer	Reverse primer
18S ribosomal RNA	18S rRNA	CCCGAAGCGTTTACTTTGAA	CCCTCTTAATCATGGCCTCA
Dopamine transporter (DAT)	Slc6a3	GCCCATTTATGCGACCTACA	GATGGTCTTTCTCAGGTGTGATG
Tyrosine hydroxylase	TH	TGTGTCCGAGAGCTTCAATG	GCTGGATACGAGAGGCATAGTTC
Glial fibrillary acidic protein	Gfap	CAACCTCCAGATCCGAGAAA	GCTCCTGCTTCGACTCCTTA
Nuclear orphan receptor (Nurr1)	Nr4a2	CGATCAGGACCTGCTTTTTG	CTGGGTTGGACCTGTATGCT
Vesicular monoamine transporter 2 (Vmat2)	Slc18a2	AGACCATGTGTTCCCGAAAG	CACATAGCCACCTTCCCATT
Glutamic acid decarboxylase 65 (GAD65)	Gad2	TTGCGAGTTCTGGAAGACAA	AGATGACCATGCGGAAGAAG
Vesicular glutamate transporter 2 (VGlut2)	Slc17a6	GATATTGCCCCGAGATATGC	CCATTCTTCACGGGACTTGT
